# Immunologic Effects of Maraviroc in HIV-Infected Patients with Severe CD4 Lymphopenia Starting Antiretroviral Therapy: A Sub-Study of the CADIRIS Trial

**DOI:** 10.20411/pai.v2i2.181

**Published:** 2017-05-09

**Authors:** Pablo F. Belaunzarán-Zamudio, Livio Azzoni, David H. Canaday, Yanink N. Caro-Vega, Brian Clagett, Mohammed S Rassool, Benigno Rodriguez, Ian Sanne, Irini Sereti, Juan G. Sierra-Madero, Michael M. Lederman

**Affiliations:** 1 Departamento de Infectología, Instituto Nacional de Ciencias Médicas y Nutrición “Salvador Zubirán”, Mexico City, Mexico; 2 División de Investigación de la Facultad de Medicina, Universidad Nacional Autónoma de México”, Mexico City, Mexico; 3 The WISTAR Institute, Philadelphia, Pennsylvania; 4 Center for AIDS Research (CFAR) Case Western Reserve University, Cleveland, Ohio; 5 Clinical HIV Research Unit, Department of Internal Medicine, Faculty of Health Sciences, University of Witwatersrand, Johannesburg, South Africa; 6 HIV Pathogenesis Section, Laboratory of Immunoregulation, National Institute of Allergy and Infectious Diseases/National Institutes of Health, Bethesda, Maryland

**Keywords:** Immune Reconstitution Inflammatory Syndrome, HIV, maraviroc, immune activation, maturation phenotype

## Abstract

**Background:**

We aimed to describe the mechanisms of immunological recovery and the effects of blocking CCR5 in patients starting ART with advanced HIV-infection.

**Methods:**

This was a sub-study of a 48 week double-blind, clinical trial where patients starting ART with CD4+ cell counts <100 cells/uL were randomized to receive maraviroc or a placebo. CD4+ and CD8+ cell maturation phenotypes, expression of PD-1 and CCR5, and activation indices were measured at weeks 0, 4, 12, 24, and 48. The reactivity of CD4+ and CD8+cells with peptides of CMV and MTb, and with Staphylococcal enterotoxin B (SEB) was assessed by intra-cellular expression of IFNγ, TNFα, and CD40 ligand at weeks 0, 4, and 12 of ART.

**Results:**

Forty patients were included in the study (Maraviroc = 22; placebo = 18). Sustained increases in CD8+ cells and in proportions of CCR5+ CD4+ and CD8+ cells were observed in the maraviroc arm. Early increases in the proportions of activated (CD38+, HLA-DR+), PD-1+ CD4+, and CD8+ cells and more matured CD8+ cells, were observed in the maraviroc arm. T cell responses to CMV, MTb, and SEB did not differ by treatment arms.

**Conclusions:**

During antiretroviral therapy in advanced HIV infection, maraviroc retains mature, activated CCR5+ cells in circulation without impact on CD4+ T cell recovery or T cell reactivity to antigen or superantigen.

**STANDFIRST**

Addition of Maraviroc to a standard ART regime increased CCR5+ and activated CD4 and CD8 cells in the circulation without affecting CD4 cell recovery or antigen-specific T-cell responses.

## INTRODUCTION

HIV-infected patients with very low CD4+ cell counts who are beginning combination antiretroviral therapy (ART) remain at risk for AIDS-defining opportunistic infections (ADEs) after ART initiation, immune response inflammatory syndrome (IRIS), and early mortality [1–6]. Increased risk of metabolic, cardiovascular, and neoplastic non-AIDS defining illnesses leading to late mortality has also been associated with low CD4+ counts at ART initiation [7–10]. The increased risk of morbid outcomes among late ART initiators appears to be related, at least partially, to the failure to achieve adequate immune recovery under virally suppressive ART [11–14].

The specific mechanisms of immunologic recovery in HIV-infected patients with advanced disease after ART initiation have not been fully characterized. During the first 4–12 weeks of ART, most of the CD4+ and CD8+ cell recovery is thought to be largely a consequence of redistribution of memory and effector cells to peripheral blood [15–17]. Later, a slow and steady increase in CD4+ cell counts is driven mostly by increasing numbers of “naive” CD4+ cells while CD8+ cells remain elevated in most patients [15–19]. These changes are accompanied by decreases in immune activation markers in memory and naive CD4+ cells, but particularly among memory CD8+ cells [17, 18, 20].

We recently conducted a randomized controlled clinical trial to ask if the CCR5-antagonist maraviroc added to an ART regimen could prevent the occurrence of IRIS. We hypothesized that CCR5 blockade would decrease immune cell migration to inflammatory tissues thereby attenuating the magnitude of clinically important inflammation. Maraviroc, which is not recommended as a first option on starting combined antiretroviral therapies, was used in addition to a recommended first line, 3-drug antiretroviral regimen to assess its immunoregulatory effect. Therefore, HIV tropism tests were not done. We enrolled 276 HIV-infected, ART naive subjects with fewer than 100 CD4+ cells/uL, and observed no difference in the emergence, severity, or timing of IRIS events between patients receiving ART plus maraviroc and those receiving ART plus placebo [21].

In this sub-study we characterized the maturation phenotype of circulating T cells and their reactivity with microbial peptides to test the hypothesis that maraviroc, by selectively retaining CCR5-expressing (CCR5+) cells in circulation, would retain both activated and antigen-specific T cells that might mediate IRIS in tissues. We found that maraviroc was associated with an early increase of CCR5+ cells in the circulation, leading to increases of peripheral activated T cells as well as more mature CD8+ cells. However, there was no increase in the proportions of T cells expressing cytokines in response to stimulation with peptides of cytomegalovirus (CMV), *Mycobacterium tuberculosis* (MTb), or in response to the superantigen Staphylococcal enterotoxin B (SEB).

## METHODS

### Study design and participants

The CADIRIS trial was a randomized, double-blind, placebo-controlled, multicenter clinical trial to assess whether maraviroc could decrease the occurrence of IRIS in HIV-infected patients beginning ART [21]. Adults were eligible if they were naive to ART, had a CD4+ cell count equal to or lower than 100 cells/μL and had plasma HIV RNA levels greater than 1,000 copies/mL. We recruited participants in South Africa and Mexico and randomly allocated them in equal proportions to receive maraviroc 600 mg twice daily or a placebo in addition to a daily standard regimen of efavirenz 600 mg, tenofovir 300 mg, and emtricitabine 200 mg. Participants were followed for 48 weeks. A detailed description of screening, accrual, treatment allocation, inclusion criteria, and follow-up procedures is available elsewhere [21]. In this sub-study, we included the first 40 patients who agreed to participate after being randomized to one of the treatment arms. Twenty patients were accrued from the Clinical HIV Research Unit, University of the Witwatersrand in South Africa and then 20 patients from the HIV/AIDS Clinic from the Infectious Diseases Department at the Instituto Nacional de Ciencias Médicas y Nutrición Salvador Zubirán, and the Hospital General de México, in Mexico. There was a separate informed consent process for this sub-study. Participation was not a condition for remaining in the parent study.

### Blood sample processing and laboratory procedures

Participants provided blood samples at baseline and after weeks 4, 12, 24, and 48 of ART. All samples were collected and processed following the *Processing Guide for the AIDS Clinical Trials Network* [22]. Briefly, we collected whole blood samples at each site in EDTA tubes and processed them within 2 hours. Plasma was retrieved in sterile cryovials and stored at -70°C. Peripheral blood mononuclear cells (PBMCs) were separated using density sedimentation and cryopre-served in 10% DMSO and 90% fetal bovine serum at -135°C on site. All samples were shipped in batch to Case Western Reserve University, Cleveland, OH, where samples were thawed and all the laboratory analyses were performed.

We performed immunophenotyping on freshly thawed PBMCs using multicolor flow cytometry. After gating for viability using Live/Dead Yellow (Invitrogen, Merelbeke, Belgium), viable cells were examined for forward and side light scatter to identify lymphocytes, and T cells were identified by their reactivity with anti-CD3^Qdot655^ (BioLegend, San Diego, CA) antibodies. The reagents to identify CD4 and CD8 cell populations, CCR5 expression, T-cell activation and maturation subsets are listed in Table 1. The T-cell maturation subsets were defined as naive (CD45RA+, CCR7+), central memory (CD45RA-, CCR7+), effector memory (CD45RA-, CCR7-) or terminally differentiated (CD45RA+, CCR7-) and T-cell activation was defined by the presence of CD38+ and HLA-DR+ markers. The T-cell subsets were examined for reactivity with anti-PD-1^BV421^ (BD BioScience, San Jose, CA) [23, 24]. The T-cell phenotyping panel was performed using a BD LSRII flow cytometer and analysis was done using BD FACSDiva software. Gating was performed using fluorochrome-conjugated isotype control antibodies.

**Table 1.** List of antibodies^a^ and reagents used for T-cell phenotyping in flow cytometry panel.

Target population	Marker	Clone	IF conjugate
T cell phenotype	CD4		BV510^b^
	CD8		AF700
	CCR5	2D7	APC-Cy7
T cell activation	CD38		BV711
	HLA-DR		FITC
T cell maturation subsets^c^	CD45RA		PE-Cy7
	CCR7		PE-CF594
T cell senescence	PD-1		BV421

^a^ All antibodies were obtained from BD BioScience, San Jose, CA.

^b^ anti-CD4^PerCP^ was used to identify CD4+ cells in functional T-cell response assays.

^c^ T-cell maturation subsets were defined as naive (CD45RA+, CCR7+), central memory (CD45RA-, CCR7+), effector memory (CD45RA-, CCR7-) or terminally differentiated (CD45RA+, CCR7-)

To analyze functional responses to stimulation, thawed PBMC were stimulated overnight with pooled CMV peptides (pp65, AIDS Reference Reagent Program), pooled peptides derived from *M. tuberculosis* (BEI Resources, Manassas, VA) [25–27], and with the superantigen staphylococcus enterotoxin B (SEB, Toxin Technology, Sarasota, FL) in the presence of brefeldin A (Golgi plug, BD Biosciences). Cells were then stained for viability using Live/Dead Violet (Invitrogen, Merelbeke, Belgium). Cells stained with Pacific Blue conjugated antibodies to CD14, CD16, and CD19 (BioLegend, San Diego, CA) were excluded with the dead cells. Surface staining was performed as indicated in Table 1. Cells were permeabilized with BD Cytofix/Cytoperm, followed by intracellular staining using anti-TNF-α ^FITC^ (BioLegend, San Diego, CA), anti-IFN-γ ^PE-Cy7^ (BioLegend, San Diego, CA), and anti-CD40L ^APC^ (BD BioScience, San Jose, CA) [28, 29] and examined using a MACSQuant flow cytometer and analyzed with MACSQuantify software.

### Statistical analysis

Patients' baseline characteristics and occurrence of IRIS events are described using mean and standard error of the mean or absolute proportions and interquartile ranges, as appropriate. We compared repeated measurements over time between the 2 treatment groups using mixed models with interactions between immune indices and time points to model and compare changes over time. We computed point estimates and 95% confidence intervals for measurements at each time point by treatment arm, and *P*-values for comparisons between treatment arms using linear combination of coefficients after model estimation. We selected the model guided by its fitness to observed data using graphic methods, and using Akaike Information Criteria. We used STATA version 11 for all statistical analyses.

### Ethical considerations

The Institutional Ethics Review Committee or Institutional Review Boards at participating sites reviewed and approved this sub-study. All patients signed a separated informed consent for the sub-study. The study was performed in compliance with the Declaration of Helsinki [30]. The CADIRIS trial is registered with Clinicaltrials.gov (NCT00988780).

## RESULTS

### Baseline characteristics and IRIS events

We included 40 patients in this study, 22 received maraviroc and 18 placebo. Participants in this sub-study did not differ at baseline from those in the larger trial, with the exception that plasma HIV levels were significantly higher in the study participants not included in the substudy (Supplementary Table 1). Baseline characteristics are summarized in Table 2. A higher proportion of patients receiving maraviroc in the sub-study developed IRIS (n = 8, 36%) than patients receiving placebo (n = 4, 22%). Twelve (32%) of the participants in the sub-study developed at least 1 IRIS event and 2 (5%) participants developed 2 IRIS events. The IRIS events and the associated pathogens are summarized in Table 3.

**Table 2.** Baseline characteristics by treatment allocation

Characteristic^a^	Maraviroc (n = 22)	Placebo (n = 18)
**Age** in years	33.9(7.1)	38.8 (10.6)
**Male,** n(%)	13 (59)	11(61)
**Race**		
Black, n(%)	10 (45)	9(50)
Mixed, n(%)	12(55)	9(50)
**Total leukocytes,** cells/uL (xl,000)	3.7(1.2)	3.2(1.1)
**CD4+ count,** cells/uL	54 (42.3)	50 (31.2)
**CD4+ percentage,** %(IQR^b^)	4.7 (2.9)	6.9(5.1)
**Nadir CD4+ count,** cells/uL	45(28)	46 (27)
**CD8+ count,** cells/uL	682 (303)	500 (264)
**CD8+ percentage,** %(IQR)	63(12.1)	60 (10.4)
**CD4+/CD8+ ratio,** mean (SD^c^)	0.08 (0.08)	0.08 (0.17)
**HIV RNA levels,** copies/mL	205,115 (345,646)	356,018 (626,518)

^a^ All characteristics are summarized as means and standard deviations except when otherwise indicated

^b^ IQR = Interquartile range

^c^ Standard deviation

**Table 3.** Immune Reconstitution Inflammatory Syndrome in trial substudy participants

Characteristics	Maraviroc (n = 22)	Placebo (n = 18)
**IRIS events, n (%)**	8(36)	4(22)
**Associated pathogen, n** (%)		
Cytomegalovirus	1(5)	-
Cryptosporidium	1(5)	-
Human Papiloma Virus	1(5)	-
Herpes simplex type 2	1(5)	-
Mycobacterium avium complex	1(5)	-
Mycobacterium tuberculosis	1(5)	1(5)
Varicella Zoster Virus	1(5)	2(10)
Herpes simplex virus	1(5)	1(5)
**Severe^1^, n(%)**	2(10)	1(5)

^1^ IRIS events were severe if they resulted in death, admission to hospital, need for invasive diagnostic or therapeutic intervention (eg, bronchoscopy, surgery, biopsy), or threatened vision.

### CD4+ and CD8+ cell count changes

**Figure 1.**
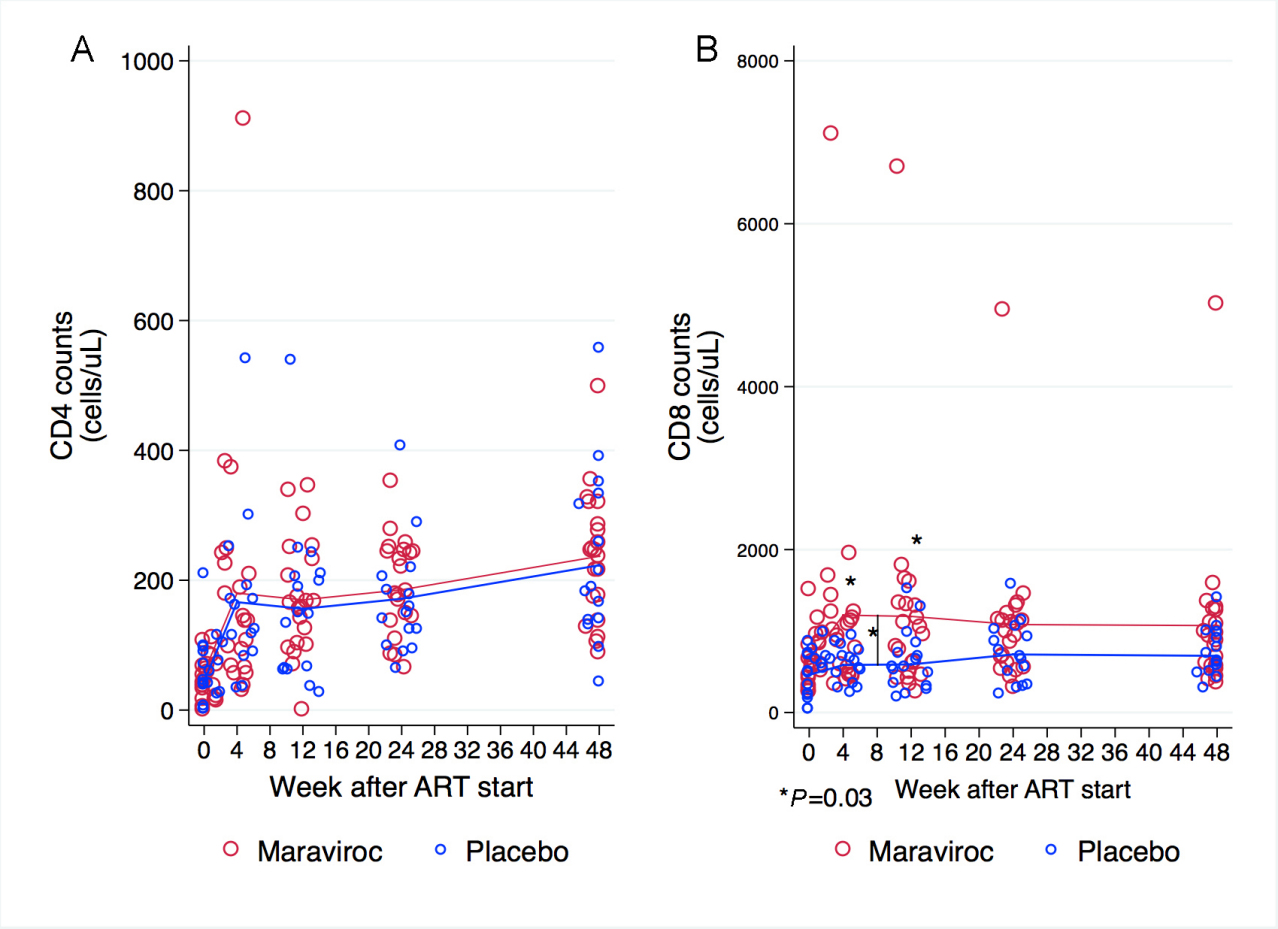
CD4+ and CD8+ cell counts at baseline, and weeks 4, 12, 24, and 48. (A) Baseline CD4+ cell counts and changes over time were similar for both groups. (B) Participants receiving maraviroc had a significantly, greater increase in CD8+ cell counts during the first 12 weeks and mean CD8+ cell counts were significantly higher at week 4 and 12. The scale of the Y-axis differs in the panels. Comparisons at each time point are indicated when *P* <0.05. The *P*-values were derived from mixed linear models using linear combination of coefficients after model estimation.

Baseline CD4+ counts and changes over time were similar for the groups (Figure 1A). There were not significant differences in mean baseline CD8+ counts by group (639 cells/uL in the maraviroc arm vs 461 cells/uL in placebo recipients, *P* = 0.5). After the first 12 weeks of treatment, the mean increase in CD8+ cell counts was greater among participants receiving maraviroc (540 cells/ uL increase from baseline) than in those receiving placebo (129 cells/uL increase from baseline) (*P* = 0.03). Mean CD8+ cell counts were significantly higher at week 4 (*P* = 0.03) and 12 (*P* = 0.03) in the maraviroc arm. Changes in CD8+ cell counts after week 12 were minimal and similar between arms. No differences were observed in CD8+ cell counts after week 8 between treatment arms (Figure 1B).

### CCR5 expression

**Figure 2.**
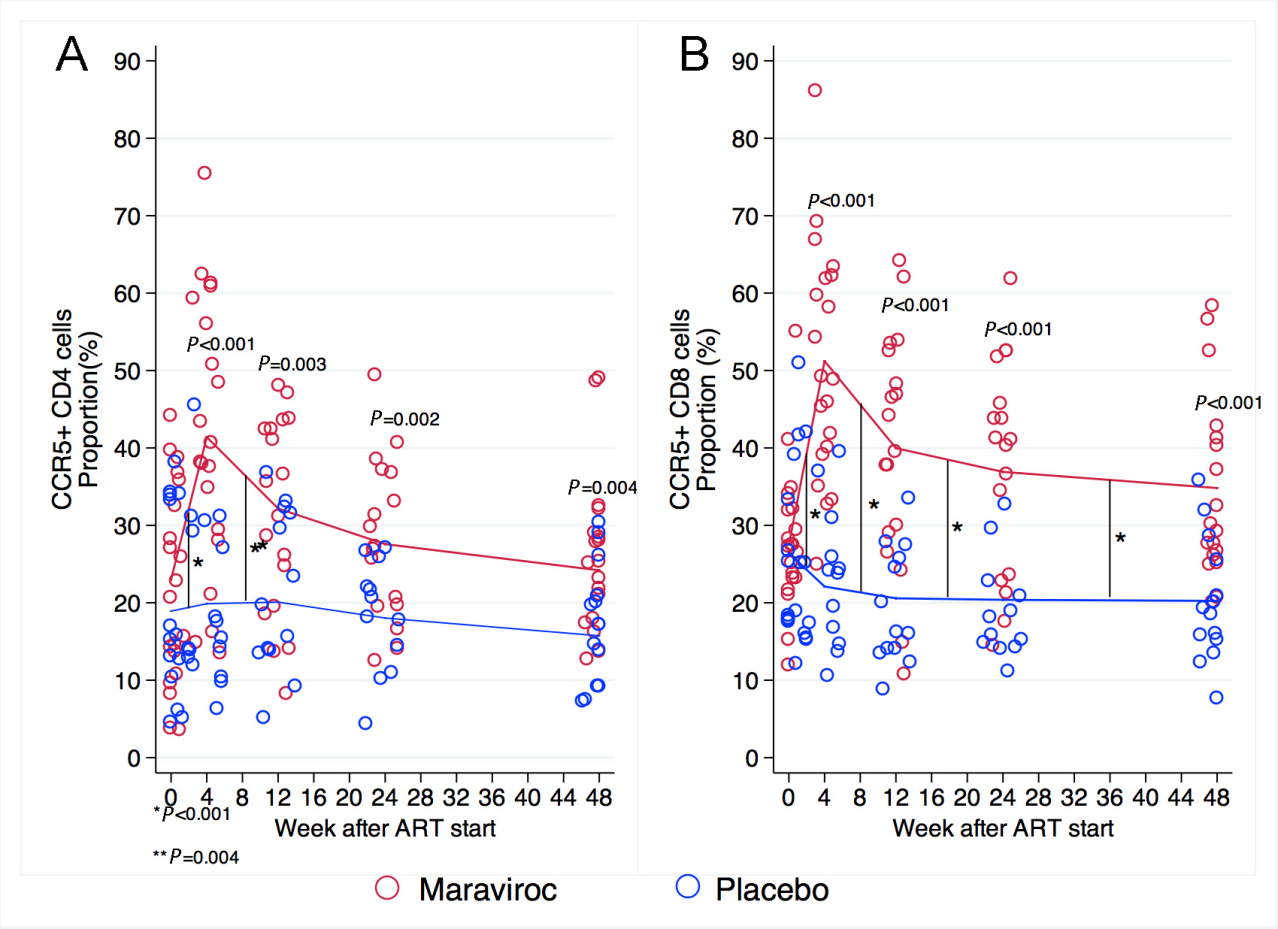
Proportion of CD4+ and CD8+ cells expressing CCR5 at baseline and weeks 4, 12, 24, and 48. (A) The proportion of CD4+ cells expressing CCR5 increased significantly during the first 4 weeks of maraviroc as opposed to the placebo group. This proportion decreased significantly in the next 8 weeks in the maraviroc arm while remaining stable in those receiving placebo. After week 12, the proportions of CD4+ cells expressing CCR5 decreased slightly in both arms. At each time point, the proportions of CD4+ cells expressing CCR5 were higher in maraviroc recipients than among placebo recipients. (B) Similarly, the proportion of CD8+ cells expressing CCR5 was not different at baseline and only increased significantly during the first 4 weeks of treatment with maraviroc. This increase peaked and fell between weeks 4 and 12 in participants receiving maraviroc and remained stable in the control group. After week 12, these proportions decreased minimally in both arms, but participants receiving maraviroc maintained higher proportions of CD8+ cells expressing CCR5 at each time point. Comparisons between treatment arms for measurements are indicated when *P*<0.05. Comparisons of the rate of change from 1 time point to the next are indicated by vertical black lines and an asterisk when *P* < 0.05. The *P*-values were derived from mixed linear models using linear combination of coefficients after model estimation.

The proportion of CD4+ cells expressing CCR5 was not different at baseline in treatment arms (18.9% in placebo vs 23% in maraviroc, *P* = 0.29). Because maraviroc is an antagonist of CCR5, we expected that the proportions of CD4+ cells expressing CCR5 would increase among maraviroc recipients, and that movement of these cells from blood to tissue compartments would be inhibited. This was shown to be the case as the proportion of CD4+ cells that expressed CCR5 increased significantly during the first 4 weeks of maraviroc but not in the placebo group (by 18.4% vs 1%, *P* < 0.001) (Figure 2). The proportion of CCR5-expressing CD4+ cells then decreased significantly between weeks 4 and 12 in the maraviroc arm while remaining stable in those receiving placebo (-9.2% *vs* 0.2%, *P* = 0.001). After week 12, the proportions of CD4+ cells expressing CCR5 decreased in both arms but not significantly. At each time point, the proportions of CCR5-expressing CD4+ cells were higher in maraviroc recipients than among placebo recipients (*P*-values for the difference between arms at each time point were < 0.001).

Similarly, the proportion of CD8+ cells expressing CCR5 was not different at baseline in the treatment arms (26.4% in placebo vs 27.5% in maraviroc, *P* = 0.8). The proportion of CD8+ cells expressing CCR5 increased significantly during the first 4 weeks of treatment on maraviroc but not on placebo (+23.7% vs -4.3%, *P* < 0.001). This increase peaked and fell between weeks 4 and 12 in patients receiving maraviroc, and CD8+ cells that expressed CCR5 decreased significantly in patients receiving maraviroc between weeks 4 and 12 but remained stable in the control group (-11.2% vs -1.5%, *P* < 0.001). After week 12, these proportions decreased minimally in both arms but not significantly. At each time point during treatment, patients receiving maraviroc had a higher proportion of CD8+ cells expressing CCR5 than did patients receiving placebo (*P*-value*s* for the difference between arms at each time point were < 0.001).

### CD4 and CD8 cell maturation subsets

**Figure 3.**
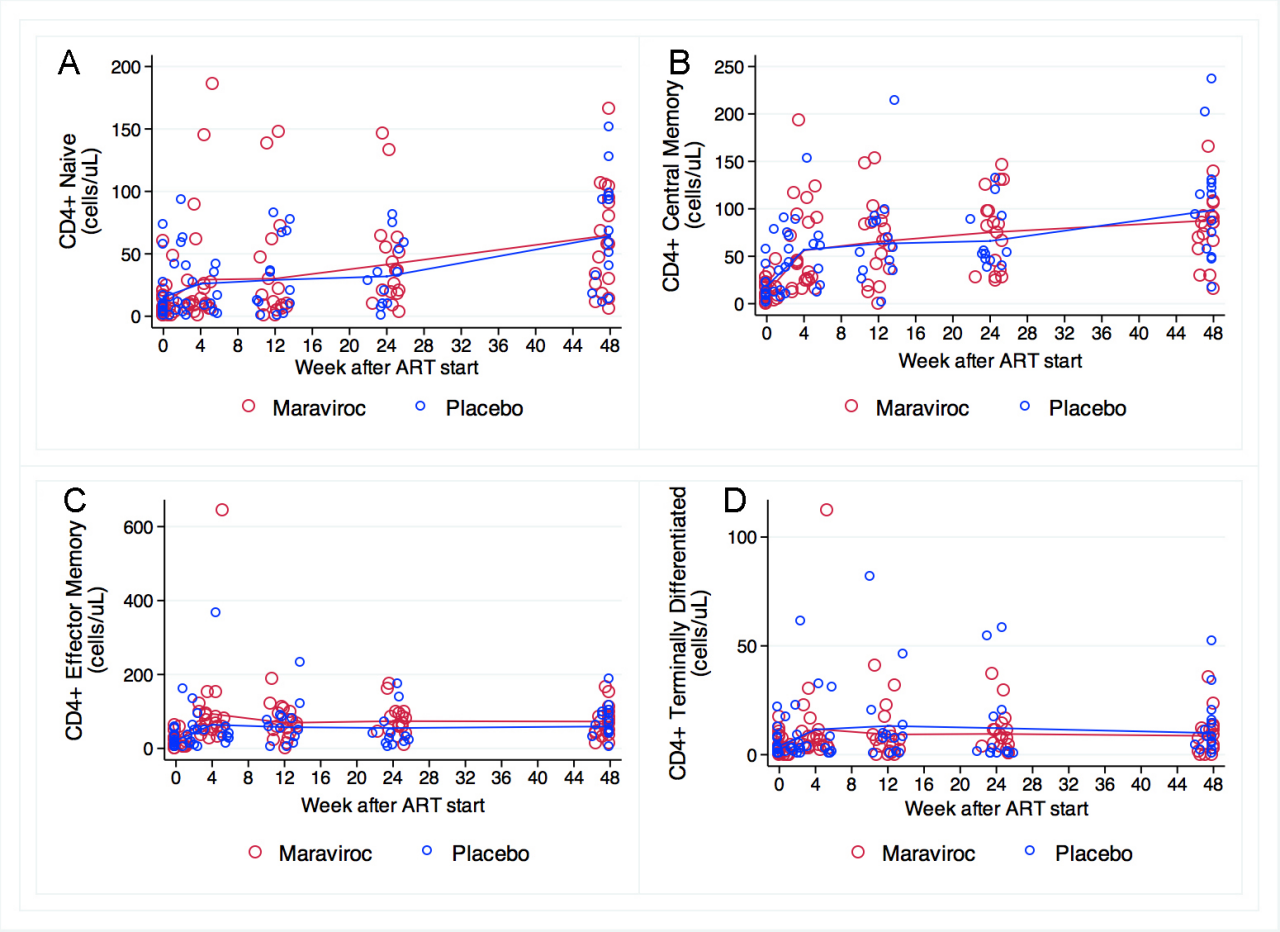
CD4 cell maturation subsets at baseline and weeks 4, 12, 24, and 48. No significant differences between groups at any time point for any CD4 maturation subtypes were observed. (A) Naive and (B) central memory CD4+ cell counts were not different at baseline and increased significantly and in parallel throughout the study in both arms. (C) Effector memory and (D) terminally differentiated CD4+ cells transiently increased in both treatment arms, and remained at similar levels until week 48 in both groups. The scale for the Y-axis differs in these panels. Comparisons of measurements and the rate of change by treatment arm were performed using mixed linear models.

Naive and central memory CD4+ cell counts were not different in the groups at baseline. In both groups, naive and central memory CD4+ cell counts increased steeply in the first 4 weeks, and then showed a slow but steady and significant increase throughout the study (Figure 3A and 3B). Effector memory and terminally differentiated CD4+ cell counts increased in both treatment arms in the first 4 weeks, but reached a plateau and remained at similar levels until week 48 in both groups (Figure 3C and 3D). There were no significant differences between groups at any time point for any CD4 maturation subtypes.

**Figure 4.**
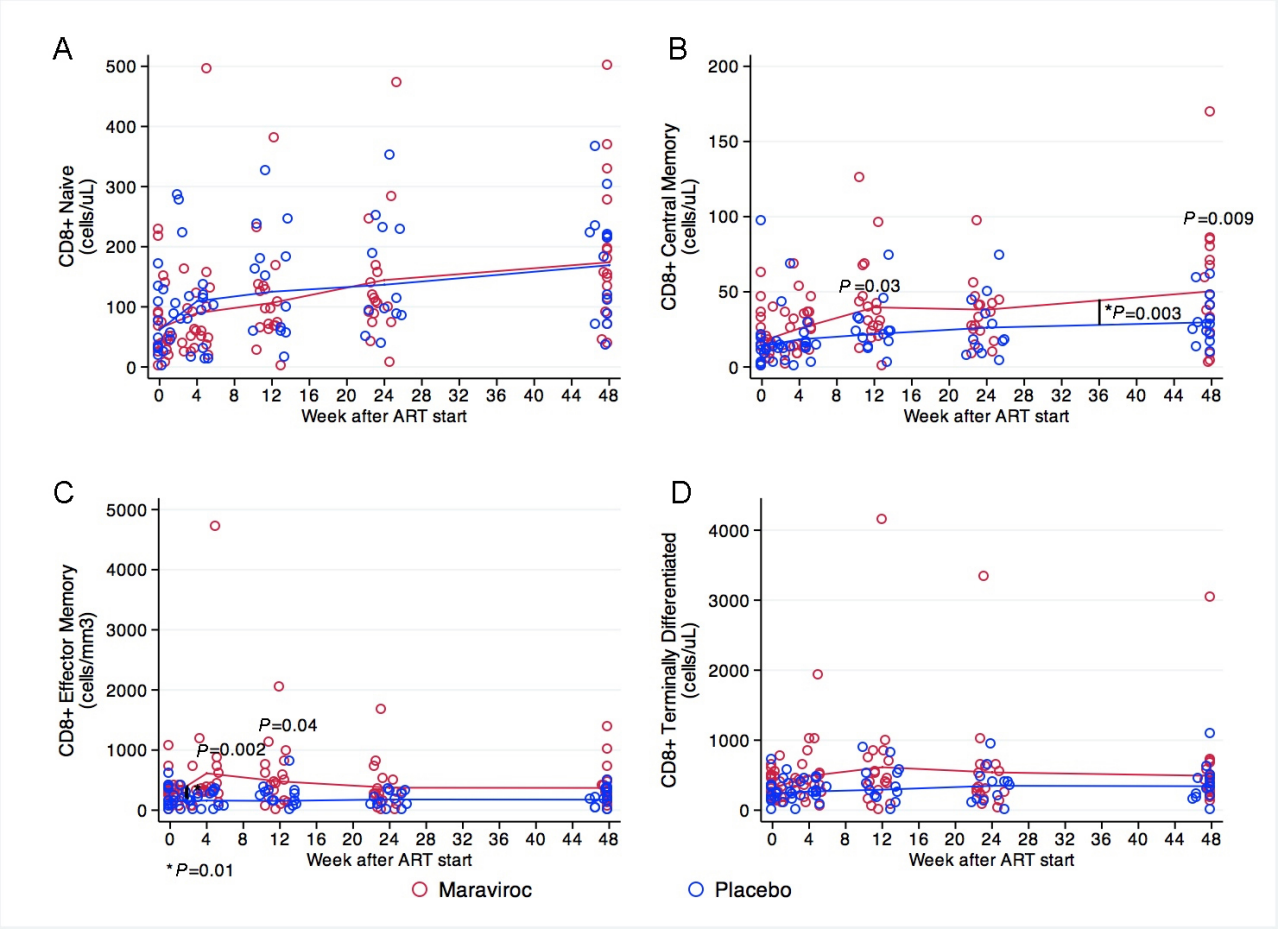
CD8+ cell maturation subsets at baseline and weeks 4, 12, 24, and 48. (A) CD8 naive cell counts steadily increased in both treatment groups. (B) Participants receiving maraviroc had a greater increase in CD8+ central memory cells during the first 12 weeks of ART in comparison to those receiving placebo. After reaching a plateau between weeks 12 and 24 in both groups, participants receiving maraviroc had greater increases in this subset, and had significantly higher counts at weeks 48. (C) Participants receiving maraviroc showed a sharp increase in CD8+ effector memory cells during the first 4 weeks of treatment, but no significant changes were observed subsequently in both groups. The CD8+ effector memory cells were significantly higher at weeks 4 and 12 in participants receiving maraviroc. (D) Terminally differentiated CD8+ cells increased in the maraviroc recipients over the 48 weeks, mostly during the first 12 weeks after treatment initiation, but no statistically significant differences between treatment arms were observed. Note that the scale for the Y-axis differs among the panels. Comparisons of mean values at each measurement between treatment arms are indicated when *P*<0.05. Comparisons in the rate of change between 1 time point to the next are indicated by a vertical line and asterisk when *P* < 0.05. The P-values were derived from mixed linear models using linear combination of coefficients after model estimation.

CD8 naive cell counts were similar at baseline in the treatment groups (*P* = 0.98) and increased steadily during the 48 weeks at similar rates in both groups. We did not observe any statistically significant differences between groups at any time point (Figure 4A). Counts of CD8+ central memory cells were not different at baseline (*P* = 0.66, Figure 4B), but patients receiving maraviroc had a greater increase during the first 12 weeks of ART (+31 cells/uL) than did those receiving placebo (+9.2 cells/ul) (*P* = 0.03). After reaching a plateau between weeks 12 and 24 in both arms, participants receiving maraviroc had a greater CD8+ central memory cell increase after week 24 (+33 cells/uL) than did those receiving placebo (+16 cells/uL) (*P* = 0.03). Patients receiving maraviroc had significantly higher CD8+ central memory cell counts at weeks 12 (40 cells/uL vs 22 cells/ul, *P* = 0.03) and 48 (50 cells/uL vs 30 cells/uL, *P* = 0.009) (Figure 4B). Effector memory CD8+ cell counts were not significantly different at baseline (*P* = 0.6). There were no changes in this subset of CD8+ cells at weeks 4, 12, 24, and 48 among patients receiving placebo. In contrast, patients receiving maraviroc showed a greater increase during the first 4 weeks (+386 cells/uL, *P* = 0.001), but these values decreased by week 12 and reached a plateau thereafter. This initial difference accounts for the significantly higher CD8+ effector memory counts among patients receiving maraviroc at weeks 4 (+452 cells/uL, *P* = 0.002) and 12 (+324 cells/uL, *P* = 0.04), but no significant differences were observed at weeks 24 and 48. (Figure 4C). Terminally differentiated CD8 cell counts significantly increased in the maraviroc recipients the first 12 weeks after treatment initiation (+306, *P* = 0.002), reached a plateau up to week 24, and decreased thereafter. In contrast, no significant changes were observed among placebo recipients at any time-point (Figure 4D).

### CD4 and CD8 cell activation markers

**Figure 5.**
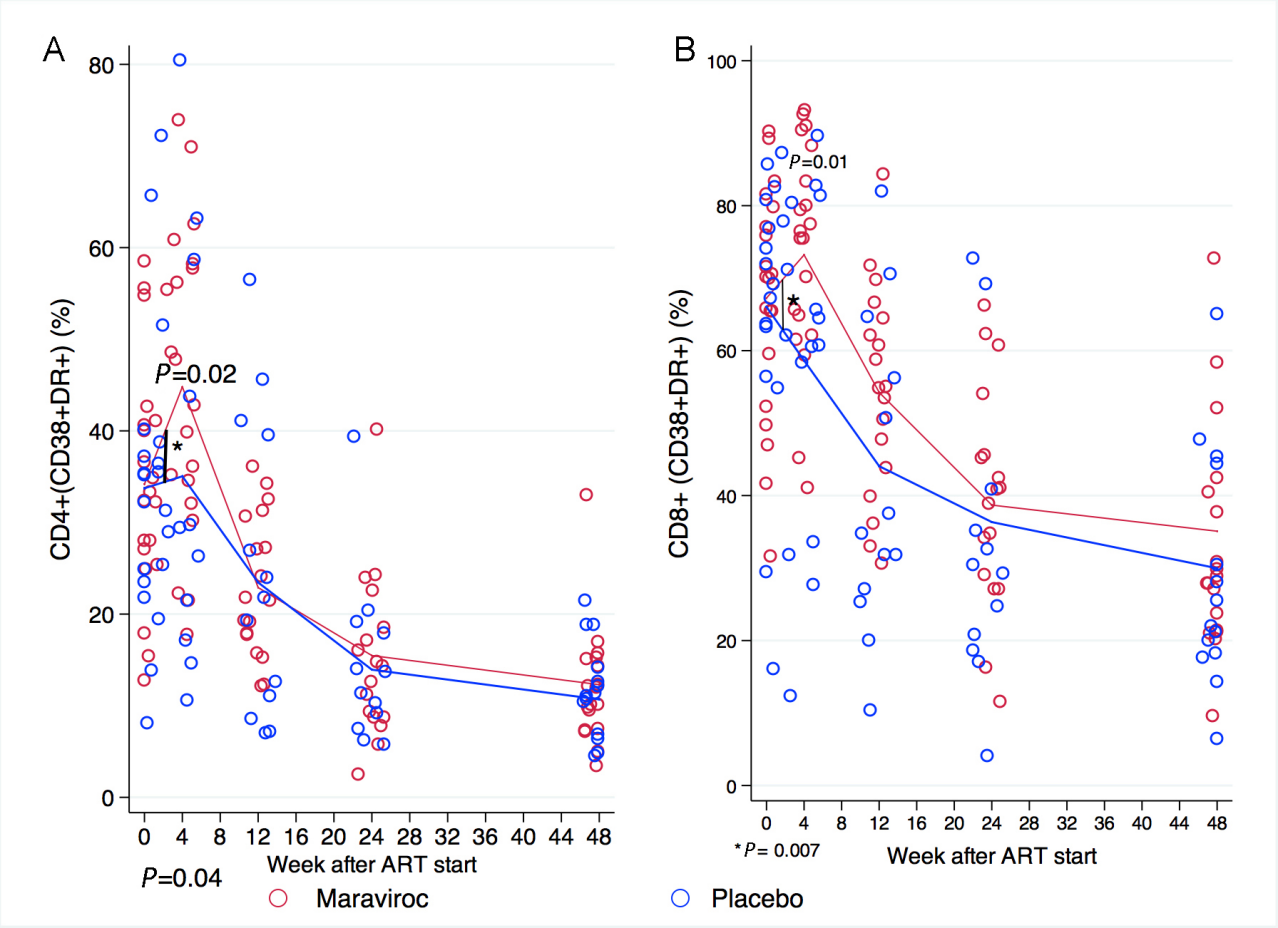
Proportion of activated (CD38+DR+) CD4+ and CD8+ cells over 48 weeks. The proportion of activated A) CD4+ and (B) CD8+ cells was similar at baseline in both groups. In the first 4 weeks, participants receiving maraviroc experienced a greater increase in the proportion of activated CD4 and CD8 cells than those receiving placebo, and had higher counts of activated CD4+ and CD8+ cells at week 4. Thereafter the proportions of activated CD4+ and CD8+ cells fell in both groups such that by week 12 and thereafter the proportions of activated CD4+ cells were similar in the treatment arms. Note that the scale in the Y-axis differs for each panel. Comparisons between treatment arms are depicted when *P* < 0.05. Comparisons in the rate of change between 1 time point to the next are indicated by a vertical line and asterisk when *P* < 0.05. The P-values were derived from mixed linear models using linear combination of coefficients after model estimation.

The proportion of activated CD4+ cells (CD38+ DR+) was similar at baseline (33.7% in the placebo arm vs 34.2% in the maraviroc arm, *P* = 0.917). During the first 4 weeks of treatment, the proportion of activated CD4+ cells increased more in patients receiving maraviroc than among those receiving placebo (by 10.6 vs 1.3%, *P* = 0.039) and reached a higher level in the maraviroc group (44.8% vs 35%, *P* = 0.02). Thereafter the proportions of activated CD4+ cells fell in both groups, such that by week 12 and after these were similar in both treatment arms. (Figure 5A)

The proportion of activated CD8+ cells (CD38+ DR+) was not different at baseline by treatment arm (66% in placebo vs 67.2% in maraviroc, *P* = 0.835). During the first 4 weeks of treatment, the proportion of activated CD8+ cells in patients receiving maraviroc increased significantly while these proportions fell in the placebo arm (+6 vs -7.4%, *P* = 0.007) such that the proportion of activated CD8+ cells at week 4 was significantly higher among patients receiving maraviroc than among those receiving placebo (73.2% vs 58.6%, *P* = 0.012). Thereafter, the activation marker frequency fell in both arms such that the proportions of activated CD8+ cells were similar in the treatment arms. (Figure 5B)

### T-cell expression of PD-1 receptor

**Figure 6.**
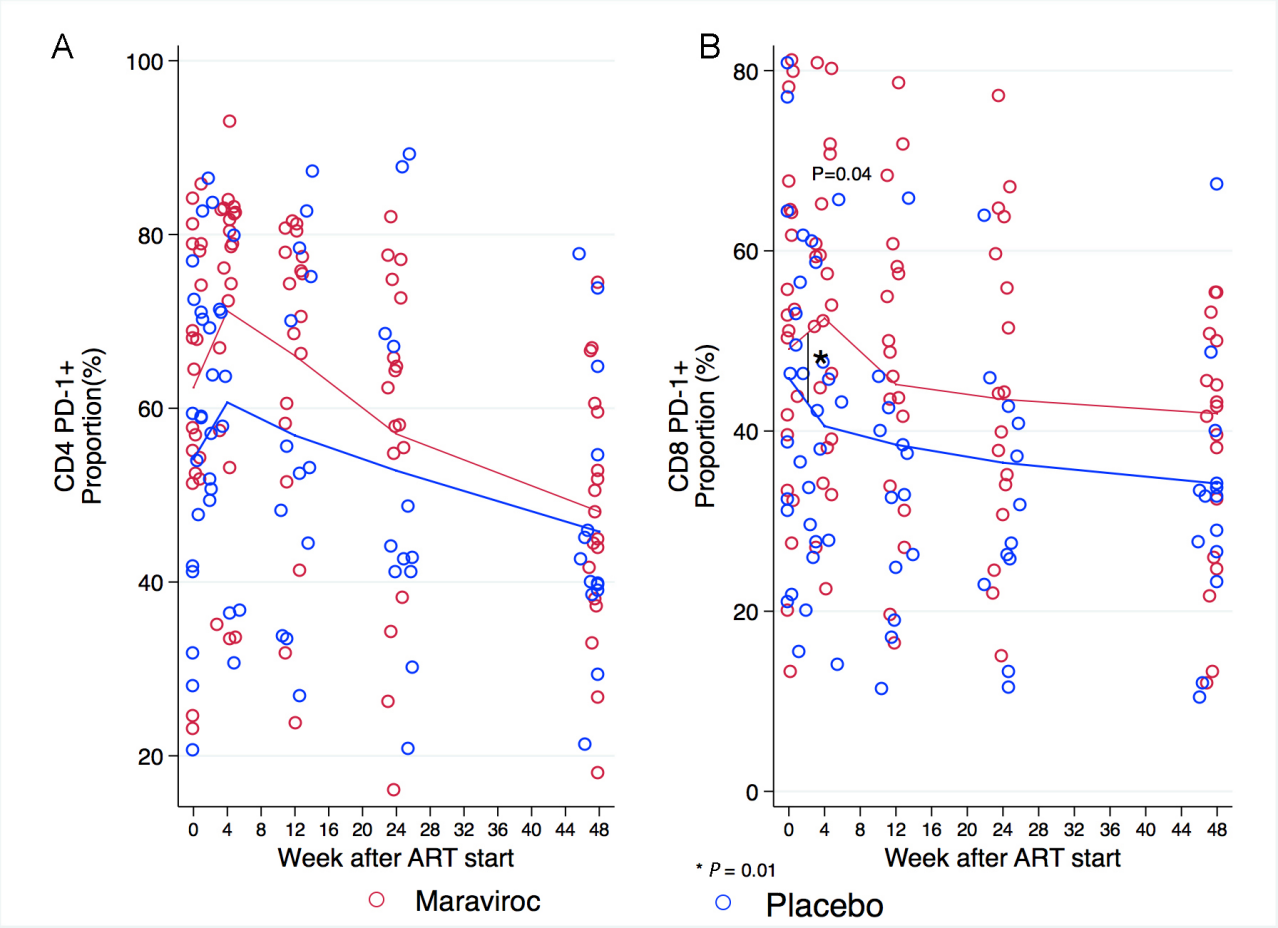
Proportion of CD4+ and CD8+ cells expressing PD-1 at baseline, weeks 4, 12, 24, and 48. The proportion of CD4+ cells expressing PD-1 increased in both arms during the first 4 weeks of treatment and then decreased throughout follow-up until the end of the study. B) The number of CD8+ cells expressing PD-1 at baseline was similar across treatment arms. The proportion of CD8+ cells expressing PD-1 was higher at week 4 in participants receiving maraviroc than in those receiving placebo. These proportions decreased in both treatment arms afterwards. Note that the scale in the Y-axis differs for each panel in the figure. Comparisons between treatment arms at measurements are depicted in the graphic when *P* < 0.05. Comparisons in the rate of change between 1 time point to the next are indicated by a vertical line and asterisk when *P* < 0.05. The *P*-values were derived from mixed linear models using linear combination of coefficients after model estimation.

The proportion of CD4+ cells expressing PD-1 increased in parallel in both treatment arms during the first 4 weeks after the initiation of ART and then decreased until the end of the study. There were no statistically significant differences in the proportions of CD4+ cells expressing PD-1 at any time point between treatment arms (Figure 6A). The proportion of CD8+ cells expressing PD-1 at baseline did not differ across treatment arms (46% in placebo vs 49% in Maraviroc, *P* = 0.6). During the first 4 weeks of treatment however, the proportion of CD8+ cells expressing PD-1 decreased in the placebo arm (5.4% decrease) but rose in the maraviroc arm (3.4% increase, *P* = 0.01). The proportion of CD8+ cells expressing PD-1 was greater at week 4 among participants receiving maraviroc than among those in the placebo group (52% vs 40%, *P* = 0.04). Between week 4 and 12, this proportion decreased more rapidly in participants receiving maraviroc than in those receiving placebo so that the proportions were similar between treatment arms at week 12 and subsequently.

### T cell functional responses

**Supplementary Figure 1.**
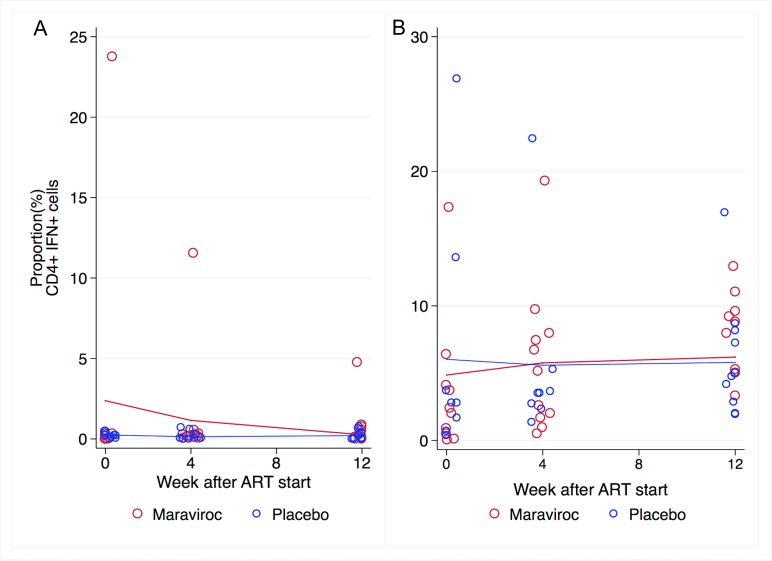
Proportion of CD4 lymphocytes expressing IFNg upon exposure to CMV peptide and SEB

**Supplementary Figure 2.**
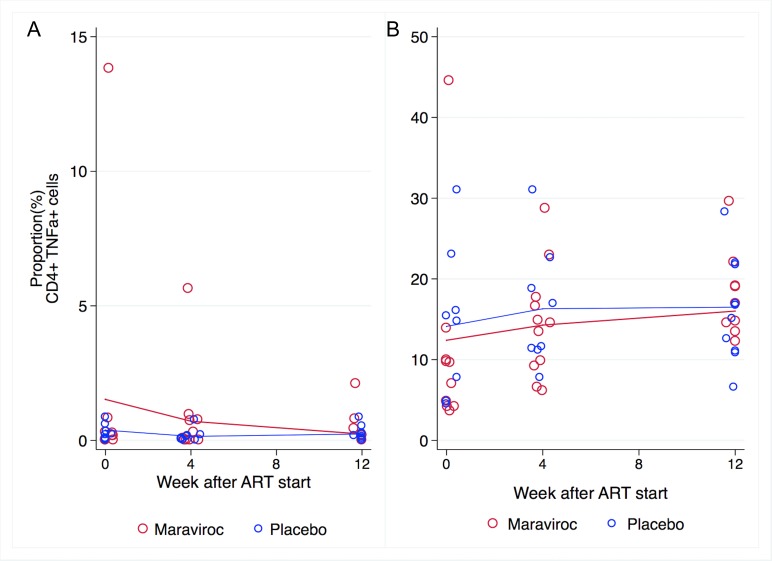
Proportion of CD4 lymphocytes expressing TNFa upon exposure to CMV peptides and SEB

**Supplementary Figure 3.**
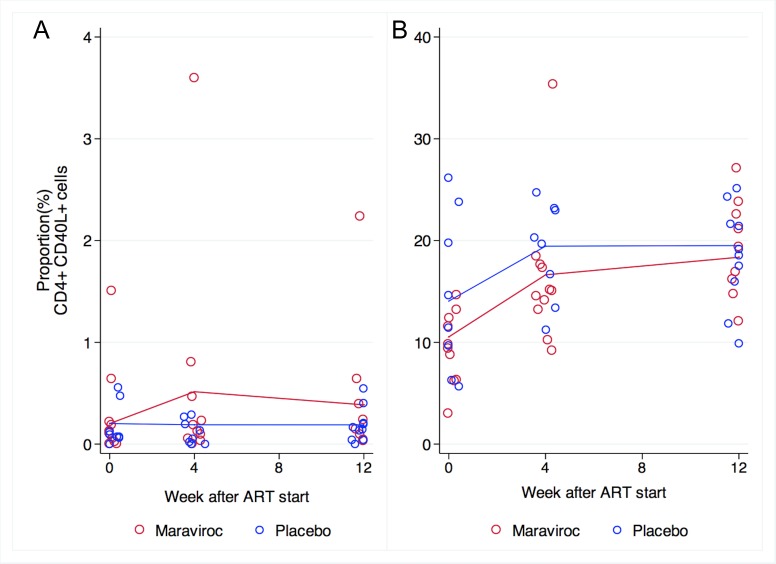
Proportion of CD4 lymphocytes expressing CD40L upon exposure to CMV peptides and SEB

The proportions of CD4+ cells expressing IFNγ or TNFα in response to CMV peptides and the super-antigen SEB were similar at baseline, week 4, and week 12 in both groups. No significant changes were observed during the 12-week observation period in any of the treatment arms (Supplementary Figure 1 and Supplementary Figure 2). Patients receiving maraviroc tended to have an increased proportion of CD4+ cells expressing CD40L after stimulation with CMV peptides at week 4 and 12, but this difference was not statistically significant (Supplementary Figure 3). The proportions of CD4+ cells expressing CD40L in response to SEB increased in both arms at week 4, and then reached a plateau. These changes were not statistically significant, nor were the small differences between treatment arms (Supplementary Figure 3).

**Supplementary Figure 4.**
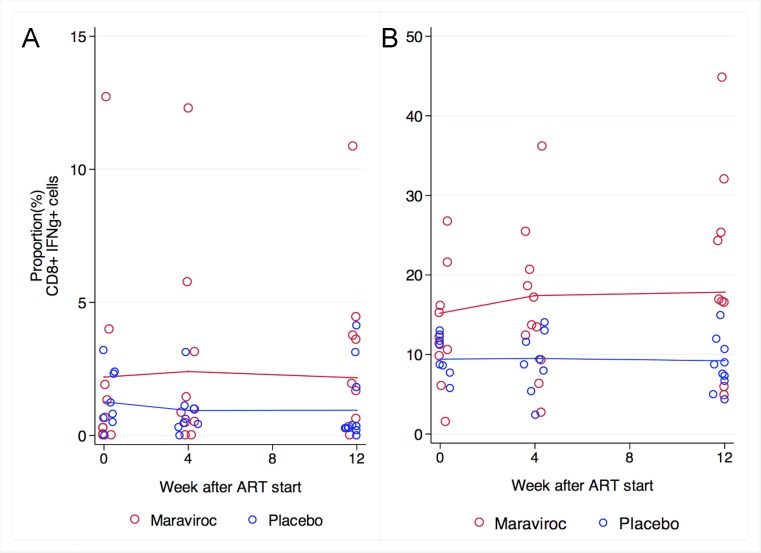
Proportion of CD8 lymphocytes expressing IFNg upon exposure to CMV peptides and SEB

**Supplementary Figure 5.**
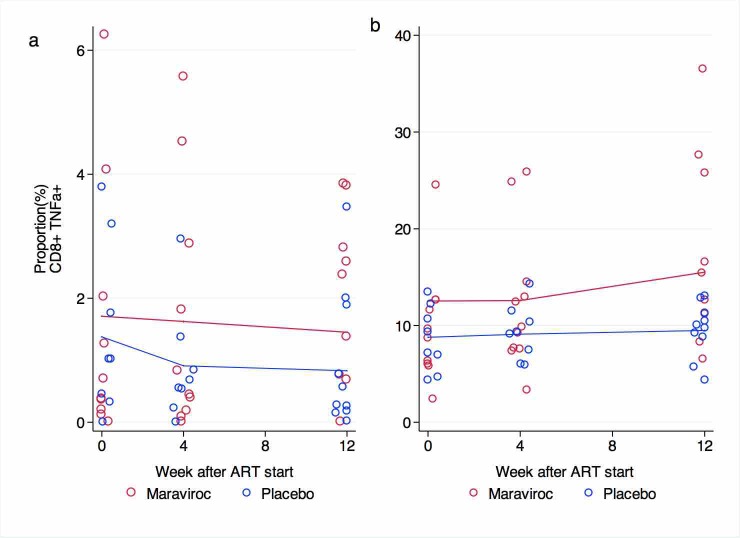
Proportion of CD8 lymphocytes expressing TNFa upon exposure to CMV peptides and SEB

The proportion of CD8+ cells expressing IFNγ after exposure to SEB tended to be higher in patients receiving maraviroc than in patients receiving placebo at baseline (15.2% *vs* 9.4%, *P* = 0.08) and remained so at week 4 (17.4% *vs* 9.5%, *P* = 0.03) and week 12 (18% vs 9.2%, *P* = 0.008). After exposure to CMV peptides, the proportion expressing IFNγ after stimulation was unchanged in both groups during the trial (Supplementary Figure 4). No significant differences at baseline or in changes in the proportions of TNFα producing CD8+ cells in response to CMV or SEB were seen between groups (Supplementary Figure 5).

## DISCUSSION

The CADIRIS trial was designed to test the hypothesis that retention of T cells in circulation by blockade of CCR5 would decrease the occurrence or severity of IRIS by preventing immune cell migration from blood into inflammatory tissues. We show here that maraviroc administration successfully retained CCR5-expressing CD4+ and CD8+ cells in circulation after initiation of a standardized combination antiretroviral therapy regimen among patients with profound immune suppression (CD4+ cells < 100cells/uL). Despite this, we observed no effect of maraviroc on the occurrence or severity of IRIS events [21]. Our findings are consistent with previous studies where maraviroc administration was associated with an increase in the proportion of CCR5+ circulating T lymphocytes [31, 32]. Presumably, this is the result of allosteric modification of CCR5, preventing chemokine binding and the subsequent internalization of the receptor [32, 33]. These data suggest that despite CCR5 blockade, other signals or other cells may be the primary drivers of tissue inflammation that is characteristic of IRIS [34].

We also observed a transient increase in the proportion of activated CD4+ and CD8+ cells in patients randomized to receive maraviroc. This may be related to the retention of CCR5-expressing cells in the circulation due to maraviroc as these tend to be more activated than cells lacking this surface receptor [35]. Nonetheless, with sustained administration of ART with or without maraviroc, T-cell activation fell as viremia resolved. Maraviroc administration also resulted in a transient increase in the proportion of CD8+ cells expressing the checkpoint inhibitor PD-1, which paralleled the transient rise in activated T cells that accompanied maraviroc administration. The functional significance of this effect is not clear. In this regard, we observed no consistent effect of maraviroc administration on CD4+ or CD8+ cell responses to peptides of CMV, *M. tuberculosis,* or to the super-antigen SEB (supplementary figures) in these patients with advanced HIV infection.

We had the opportunity to examine the effects of ART or ART plus maraviroc on the restoration of T- cell maturation subsets. Among circulating CD4+ cells, subsets of naive, central memory, effector memory, and terminally differentiated cells rose in parallel in the 2 treatment groups. Among ART plus placebo recipients, absolute numbers of CD8+ cells remained stable over the 48-week course of treatment. In contrast, administration of ART plus maraviroc resulted in an early, significant increase in absolute numbers of CD8+ cells that were higher than among ART-alone recipients at weeks 4 and 12. It is not surprising that absolute numbers of more differentiated CD8+ cells (central memory and effector memory) were greater during these early periods among maraviroc recipients as these more mature CD8+ cells are often CCR5+ and thus appear to be retained in circulation. The clinical relevance of this increase is uncertain, and this increase in mature presumably antigen-experienced cells was not reflected in an increase in the inducible expression of inflammatory cytokines by CMV or MTb peptides.

The detailed immune phenotyping performed in this study is, to our knowledge, the first analysis of the immunomodulatory effects of the CCR5 inhibitor maraviroc that largely controls for the effects of antiretroviral therapy. In the parent study, there was no demonstrable additive effect of maraviroc on indices of virologic control [21]. On the other hand, the intensive monitoring reported here was performed on only a subset of 40 participants of the main study. Thus, subtler effects of maraviroc administration might have been missed. Nonetheless, we could demonstrate the apparent retention of CCR5+ T cells, particularly more mature CD8+ cells in the circulation of patients receiving maraviroc. Similarly, maraviroc resulted in greater percentages of circulating monocytes expressing CCR5 in these participants [36]. These data also confirmed that the failure of this intervention to affect the occurrence or severity of IRIS events is not related to a failure of maraviroc to block CCR5 function by retaining CCR5+ cells in circulation. By chance, in this sub-study (but not in the main study) more patients assigned to maraviroc (8/22) than assigned to placebo (4/18) experienced clinically defined IRIS. The occurrence of IRIS did not affect the immunologic differences reported here, because findings were similar when only results among subjects who did not experience IRIS were analyzed (data not shown).

In individuals with advanced HIV infection beginning a combination antiretroviral therapy regimen, CCR5 blockade with maraviroc effectively retains CCR5+ T cells, particularly antigen-experienced CD8+ cells, in the circulation without impact on the severity or occurrence of IRIS. Thus, ligation or activation of CCR5 is not an important contributor to the occurrence of IRIS.

## SUPPLEMENTARY MATERIALS

This sub-study was designed to better understand the mechanisms of immunological recovery in HIV-infected patients starting antiretroviral therapy (ART) with very advanced disease; and to observe the immune effects of a CCR5 inhibitor to a standard ART regime under the hypothesis that CCR5 blockade would prevent the occurrence and severity of IRIS by decreasing immune cell migration to inflammatory tissues. We found that, when added to a standard ART regimen in advanced AIDS, maraviroc led to higher CCR5+ CD4 and CD8 cells in circulation without affecting antigen-specific T-cell responses. Sustained increases in circulating CD8+ cell counts were observed in patients receiving maraviroc. This document contains tables and graphics supplementary to the main results.

**Supplementary Table 1.**

Characteristic	Sub study A (n = 40)	No sub study A (n = 36)	*P*-value
**Age in** years, mean(SD)	36.07(9.07)	37.29(8.62)	0.41
median(IQR)	35.5(28.2-43.5)	36(31-43)	0.71
**Male,** n(%)	24(60)	154(65.25)	0.52
**Hemoglobin,** mg/dL	12.09(1.79)	12.16(1.77)	0.81
**White blood cells,** cells/dL(xl,000)	3.47(1.18)	3.65(1.42)	0.42
**CD4 count,** cells/μL	51.92(37.34)	44.12(41.11)	0.26
**CD4 percentage,** %(IQR)	5.70(4.11)	4.97(5.49)	0.42
**Nadir CD4 count,** cells/μL	45.32(27.09)	41.41(25.13)	0.37
**CD8 count,** cells/μL	600.37(296.93)	619.27(366.40)	0.75
**CD8 percentage,** %(IQR)	61.79(11.34)	62.13(12.07)	0.86
**HIV RNA measurement,** copies/mL	273,021(491,133)	553,262(905,790)	0.03
**Race**			
Asian, n(%)	**-**	2(0.8)	
Black, n(%)	19(47.5)	127(53.8)	
Mixed, n(%)	21(52.5)	93(39.4)	0.24
White, n(%)	**-**	14(5.9)	

Participants in this sub-study did not differ at baseline to those in the larger trial, with the exception that plasma HIV levels were significantly higher in the study participants not included in the sub-study All characteristics are summarized using means and standard deviations except when indicated.
